# Supplementary figures and images for: Relaxation enhancement by microwave irradiation may limit dynamic nuclear polarization
Source: Phys Chem Chem Phys. 2024 Feb 7;26(12):9578–85. doi: 10.1039/d3cp06025j (PMC10954235; doi:10.1039/d3cp06025j)

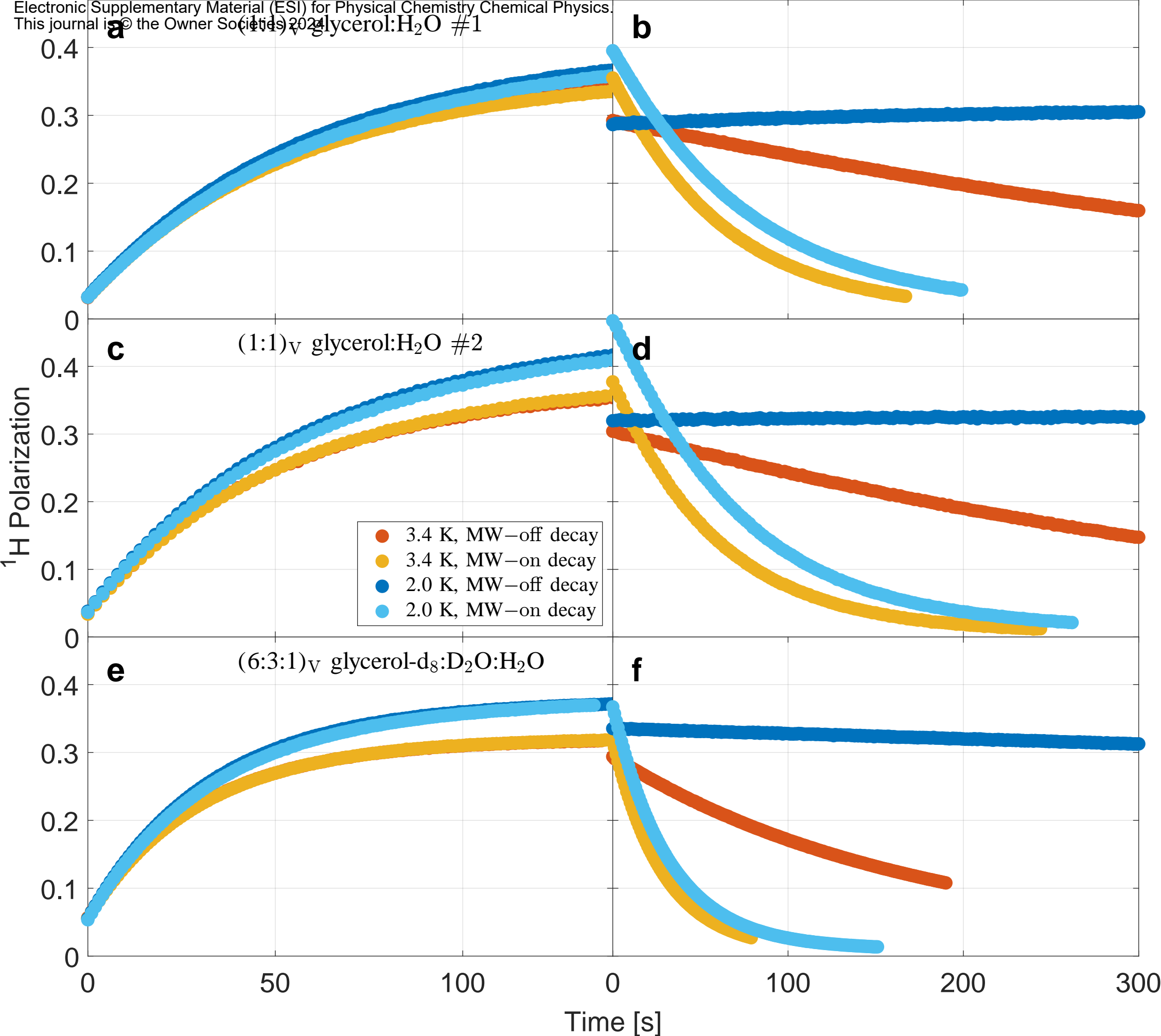

Supplement: CP-026-D3CP06025J-s001 [file CP-026-D3CP06025J-s001.pdf]

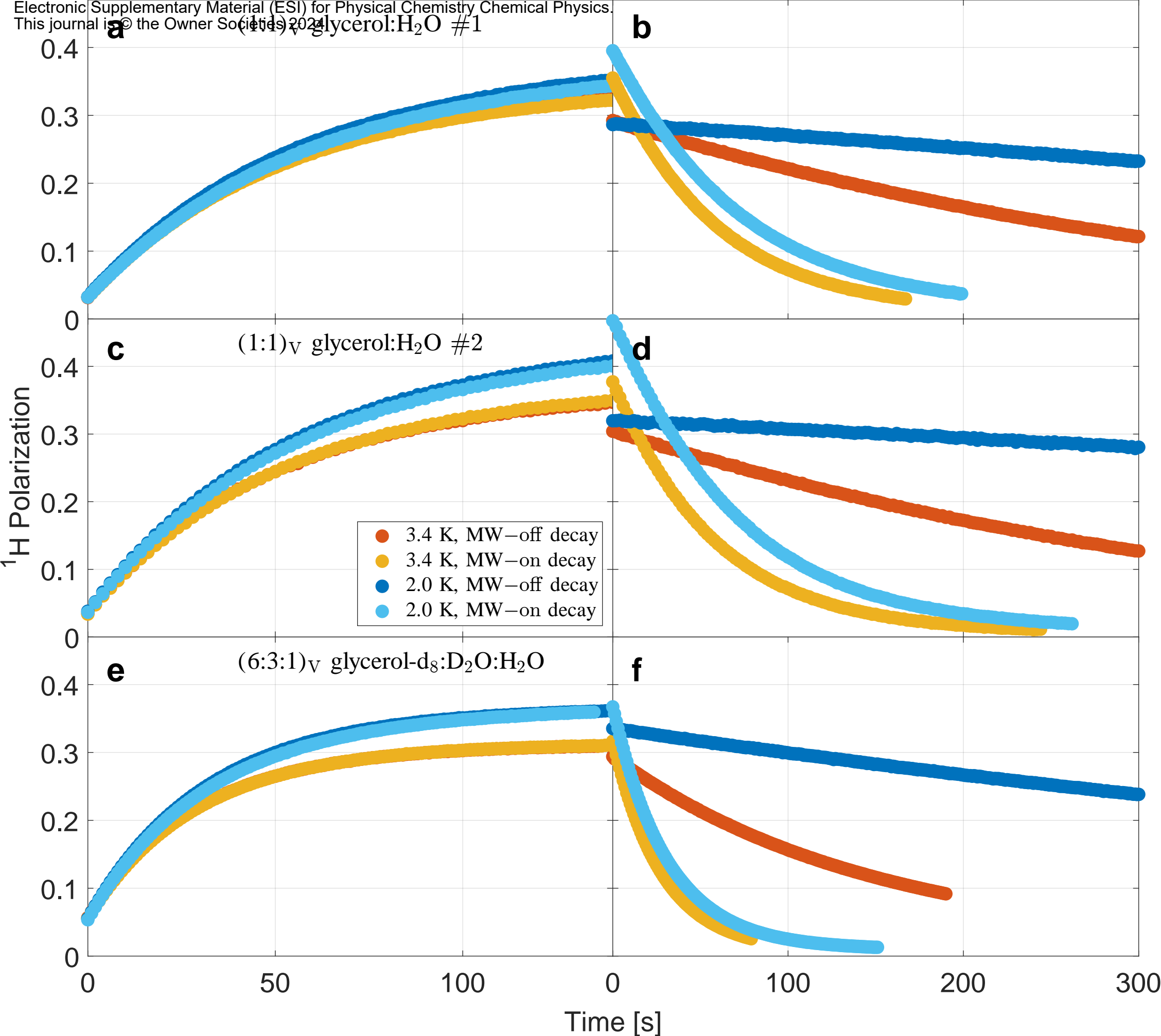

Supplement: CP-026-D3CP06025J-s002 [file CP-026-D3CP06025J-s002.pdf]

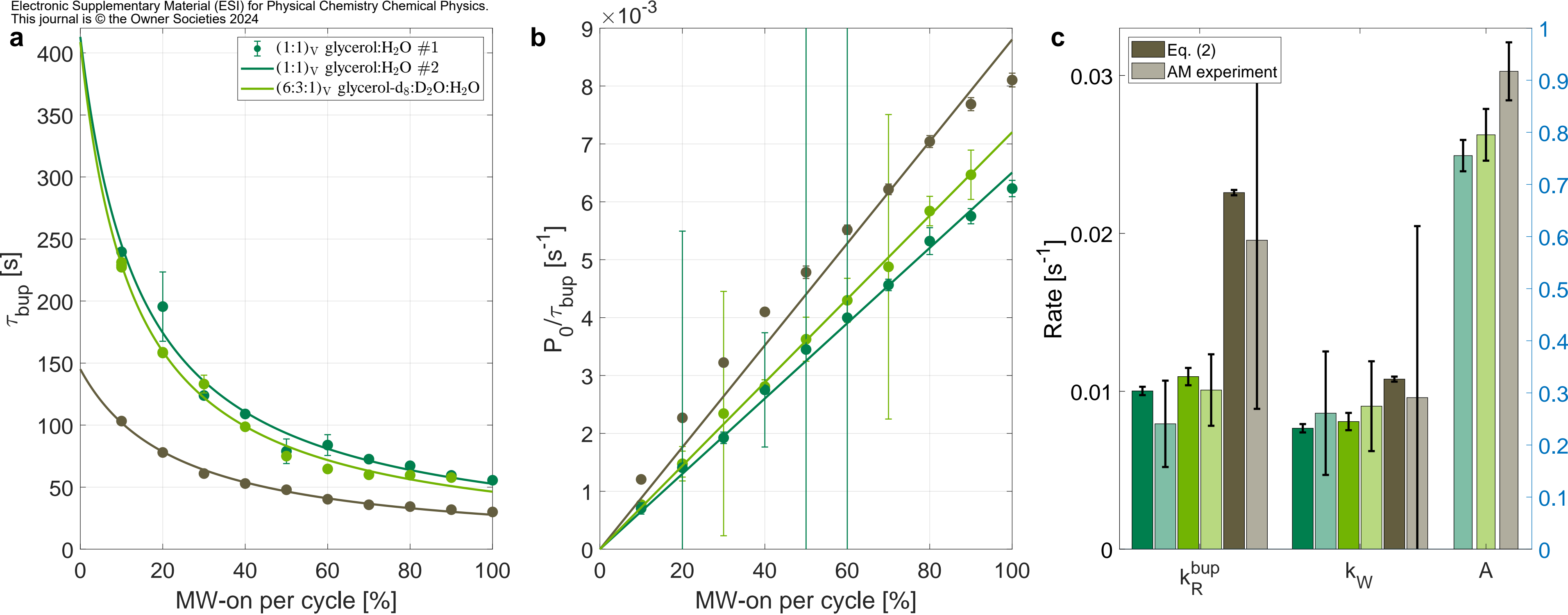

Supplement: CP-026-D3CP06025J-s003 [file CP-026-D3CP06025J-s003.pdf]

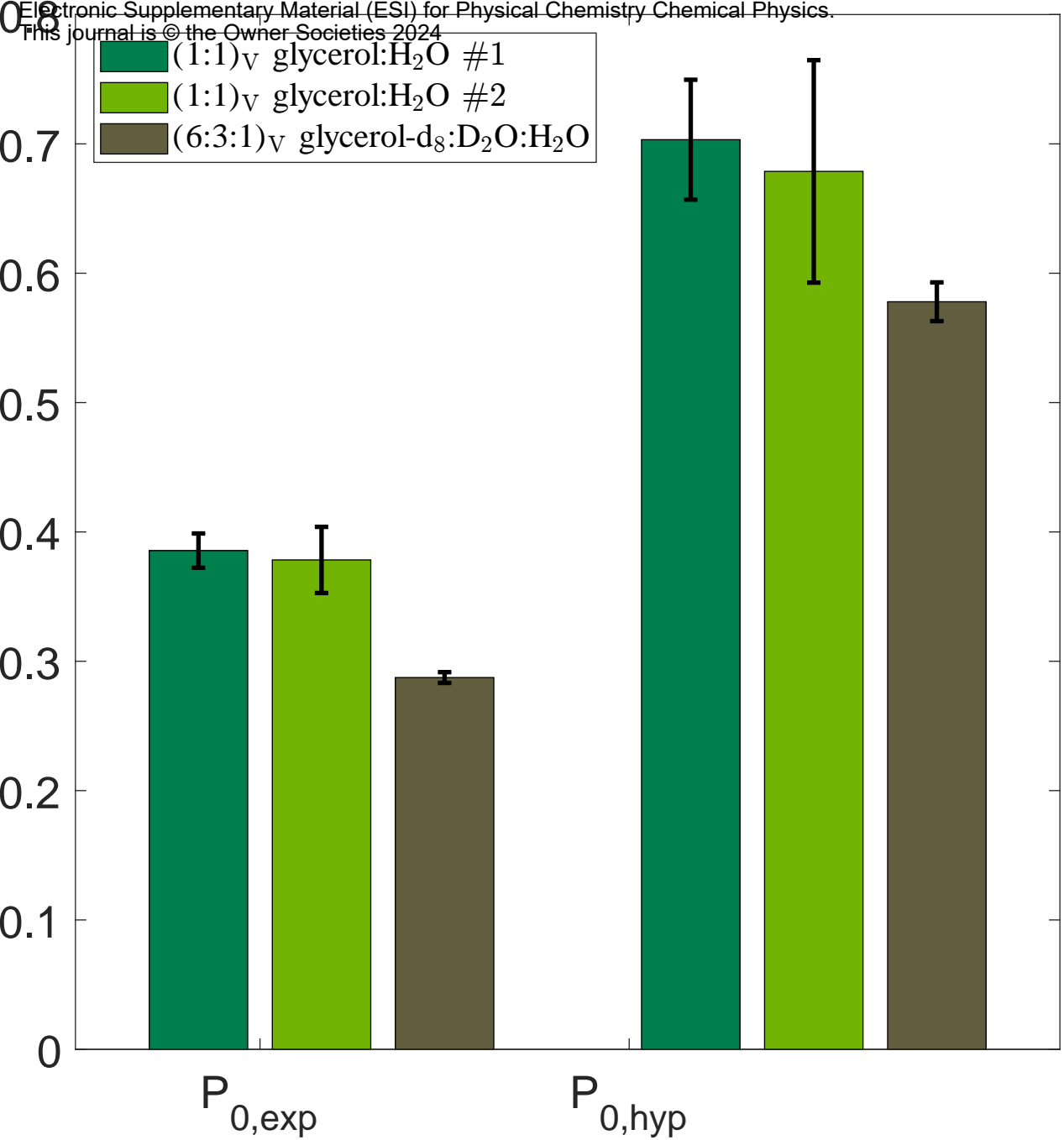

Supplement: CP-026-D3CP06025J-s004 [file CP-026-D3CP06025J-s004.pdf]
