# Supplementary material for: Relaxation enhancement by microwave irradiation may limit dynamic nuclear polarization
Source: Phys Chem Chem Phys. 2024 Feb 7;26(12):9578–85. doi: 10.1039/d3cp06025j (PMC10954235; doi:10.1039/d3cp06025j)
Supplement: CP-026-D3CP06025J-s005 [file CP-026-D3CP06025J-s005.pdf]

Electronic Supplementary Material (ESI) for Physical Chemistry Chemical Physics.
This journal is © the Owner Societies 2024

Supplementary Information: Relaxation enhancement by microwave irradiation may limit dynamic nuclear polarization

Gevin von Witte,[a,b] Aaron Himmler,[b] Sebastian Kozerke,[a] and Matthias Ernst[b]*

[a] *Institute for Biomedical Engineering, University and ETH Zurich, 8092 Zurich, Switzerland*
[b] *Department of Chemistry and Applied Biosciences, ETH Zurich, 8093 Zurich, Switzerland*
* *Corresponding author: maer@ethz.ch*

Contents

S1 Build-up and decay data with and without RF correction 2
S2 Experimental validation of the one compartment model 4
S3 Hypothetical polarization 6
S4 Measurement of the MW frequency dependence of the relaxation enhancement 6
S5 Sample heating 7

S1 Build-up and decay data with and without RF correction

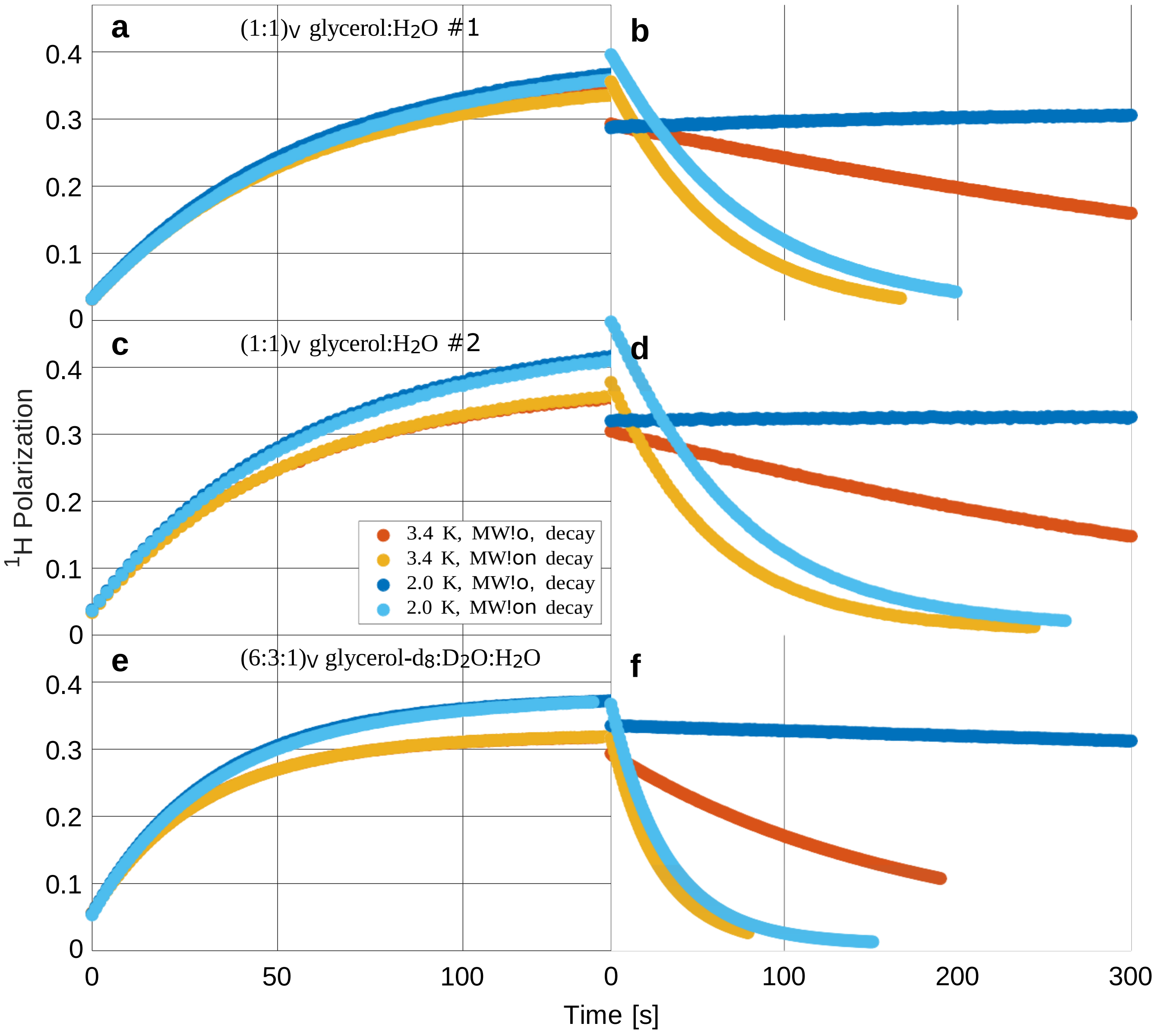

Figure S1: Build-up and decay with RF correction Build-ups (a) and the respective decays (b) at 3.3 K (red and orange) and 2.0 K (light and darker blue) with correcting for the effects of radio-frequency (RF) pulses. The MW is set to the same power and frequency (197.05GHz) during all build-ups. During the darker decays (darker blue and red), the MW is switched off. For the lighter decays (3.3 K in orange and 2.0 K in light blue), the MW is switched to the central EPR frequency (197.28 GHz) resulting in zero DNP, as displayed in Fig. 1d.

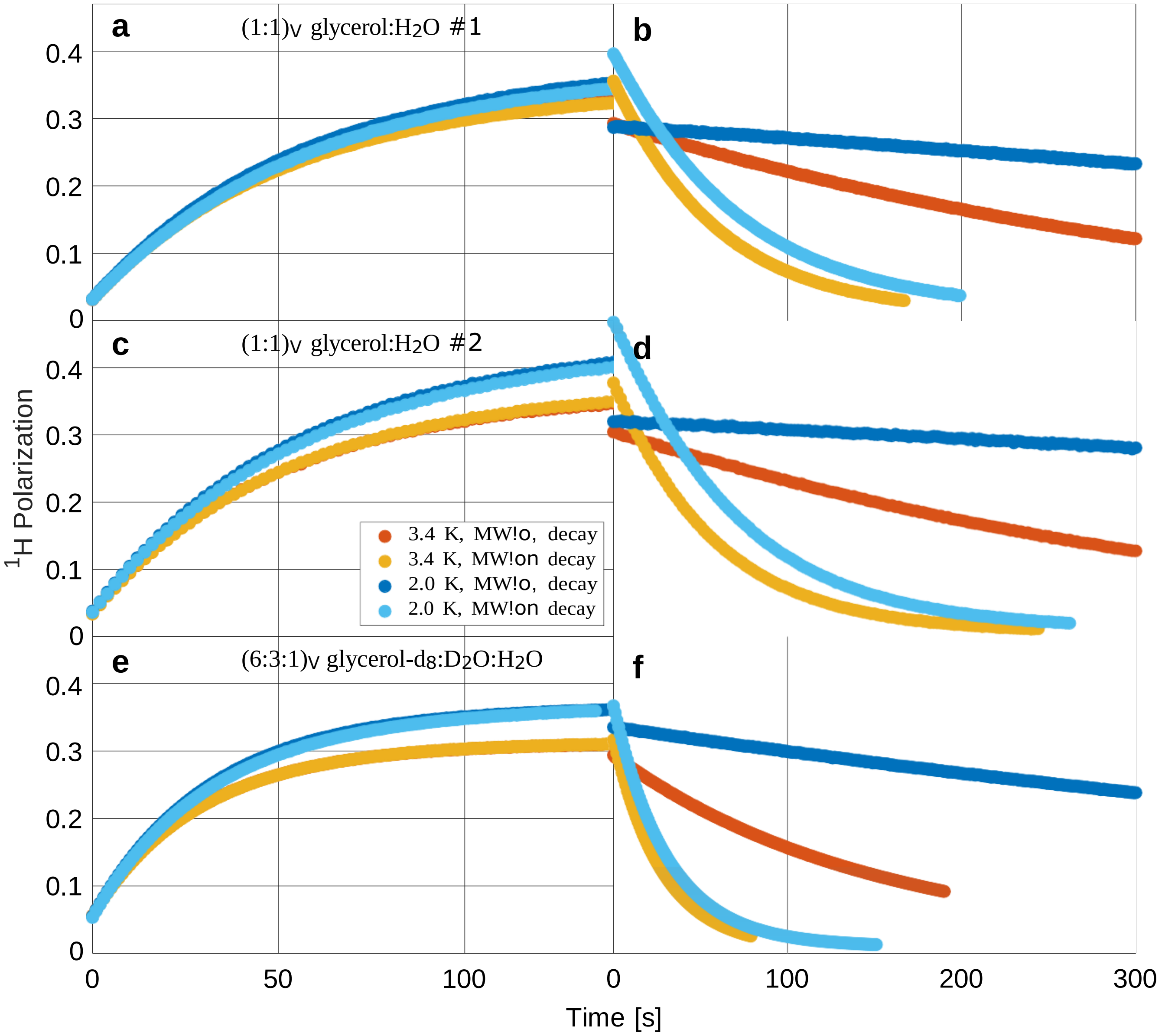

Figure S2: Build-up and decay without RF correction Build-ups (a) and the respective decays (b) at 3.3 K (red and orange) and 2.0 K (light and darker blue) without correcting for the effects of radio-frequency (RF) pulses. The MW is set to the same power and frequency (197.05GHz) during all build-ups. During the darker decays (darker blue and red), the MW is switched off. For the lighter decays (3.3 K in orange and 2.0 K in light blue), the MW is switched to the central EPR frequency (197.28 GHz) resulting in zero DNP, as displayed in Fig. 1d.

S2 Experimental validation of the one compartment model

In order to verify the validity of the extracted model parameters, namely k_W and k_{R}, from our measurements, we performed build-up experiments with amplitude modulation. In our case, we switched the MW between zero and full output power in a fixed cycle. For the choice cycle time, we have two different time scales to consider: (i) It should be much longer than the thermal $T_{1,e}$ (around 500 μs under our DNP conditions measured with longitudinal detected (LOD) electron paramagnetic resonance (EPR) [1], probably dominated by electron spectral diffusion) such that we can consider the MW irradiation blocks as continuous wave irradiation and (ii) much shorter than the build-up time such that we can average our modulated irradiation over time and consider it to be a constant irradiation on the time scale of the build-up. Based on these consideration, we chose a cycle time of 1 s. This would even be enough if the LOD EPR-based $T_{1,e}$ is two orders of magnitude shorter than the "true" $T_{1,e}$. Upon varying the time with full MW output power per cycle, we can translate this into a linear variation of our model parameters, eventually enabling us to disentangle the MW-induced relaxation from the DNP injection.

For the amplitude modulated build-ups (MW switched between full and zero power) as shown in Fig. S3, the large error bars are a result of electronics communications problems leading to clear bumps in the data (not shown), visible during processing. The affected data sets were cut but often resulted in short, usable build-up durations, leading to relatively large uncertainties in the fit results.

The estimated values of the thermal electron polarization (A) appear below the thermal electron polarization based on the experimental conditions (89% at 3.3 K and 7 T) which might be an indication of sample heating from MW absorption as discussed in the main text.

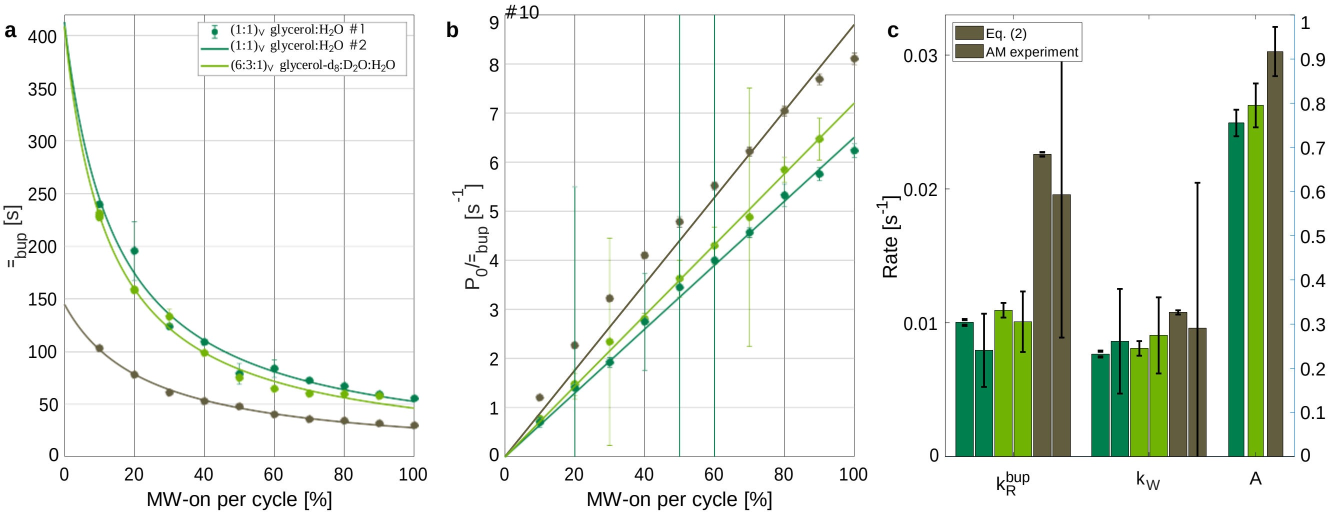

Figure S3: Measuring the one compartment model parameters. (a) We fit the build-up times for different fractions of the MW being switched on to full power (amplitude modulation with full and zero power) with $(k_{R,\mathrm{res}} + b \cdot x)^{-1}$. $k_{R,\mathrm{res}}$ is a fitted residual relaxation rate (in good agreement with the thermal relaxation rate in MW-off decays, see Fig. 1c or [2], $b = k_W + k_{R,\mathrm{MW}}$ and x being the MW-on fraction. The green data sets belong to natural abundance and brown-gray (taupe) to the partially deuterated sample as throughout this paper. (b) P_0/τ_{bup} shows a linear behaviour as expected from Eq. 2b with the slope being given by Ak_W. (c) Comparison of k_R^{bup} and k_W through two different analysis approaches: (i, full color) Inserting the measured steady-state polarization and build-up time into Eqs. 2, (ii, half transparent) The relaxation rate k_R^{bup} can be estimated from the fit shown in Fig. 2a. With k_R^{bup} at hand, k_W can be extracted from the fit shown in part (a) of this figure. Finally, A can be deduced from the linear fit shown in (b).

S3 Hypothetical polarization

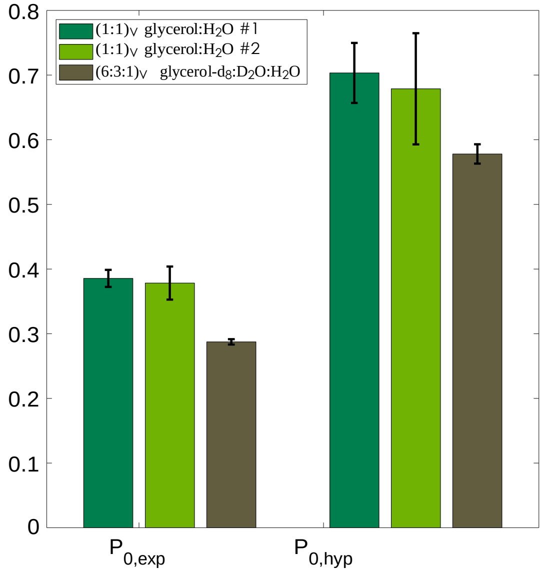

Figure S4: Experimental and hypothetical polarization. The experimentally measured steady-state polarization $P_{0,\mathrm{exp}}$ of the three samples is compared with a hypothetical polarization $P_{0,\mathrm{hyp}}$ without MW-induced relaxation (see thermal relaxation in Fig. 1c). This hypothetical polarization was calculated with Eq. 2b, the thermal electron polarization $A = 0.89$ and k_W extracted from the measured build-up time and steady-state polarization (compare Fig. S3c).

S4 Measurement of the MW frequency dependence of the relaxation enhancement

During the measurement it needs to be carefully checked that the chosen MW frequency during the decay does not lead to a significant hyperpolarization as the measured relaxation time in these types of decay is the build-up time. Only for one of the presented data points (196.75 GHz), a build-up with signals clearly exceeding the thermal signal could be measured with the steady-state polarization being less than ten percent of the used build-up frequency (197.05 GHz), justifying the usage of the measured decay time as a relaxation rate.

S5 Sample heating

Sample heating due to MW absorption would appear (nearly) frequency independent over the narrow experimental frequency regime [3] and might explain the enhanced relaxation for far off-resonant MW irradiation (see Fig. 2a) compared to the MW off situation. An upper limit for the MW absorption by the sample can likely be estimated with the imaginary index of refraction κ for ice reported in Ref. [3]: We choose $\kappa \approx 10^{-2}$ to include a potential stronger increase at liquid helium temperature, the presence of glycerol and TEMPO as well as deuteration in our experiments. With the absorption coefficient $a = 4\pi\kappa/\lambda$, our MW power at the sample space of 65 mW and a one centimeter long sample container (inner diameter three millimeter, giving around 100 mg of sample mass), would give a heating power around 37 mW. The density of ice is around 935 kg [4]. However, the specific heat of ice appears strongly temperature dependent below 100 K and the lowest temperature in Ref. [4] is around 12 K. Using this likely way too large value for the specific heat, the absorbed MW power would warm the sample by around 18 K/s in the absence of cooling. It appears possible that the bulk frozen sample within the sample container reaches a temperature exceeding the surrounding helium bath as cooling is insufficient to fully compensate for the absorbed MW power. An increase in temperature then causes increased paramagnetic relaxation.

Such a sample heating would be consistent with LOD EPR detected sample heating (chapter 5 of [5]) under conditions similar to the DNP experiments in this work (3.4 K, 7 T identical to measurements in this work but larger sample container).

References

[1] Aaron Himmler, Mohammed M Albannay, Gevin Von Witte, Sebastian Kozerke, and Matthias Ernst. Electroplated waveguides to enhance DNP and EPR spectra of silicon and diamond particles. *Magnetic Resonance*, 3(2):203–209, 2022.

[2] Gevin von Witte, Matthias Ernst, and Sebastian Kozerke. Modelling and correcting the impact of RF pulses for continuous monitoring of hyperpolarized NMR. *Magnetic Resonance*, 4:175–186, 2023.

[3] Stephen G. Warren and Richard E. Brandt. Optical constants of ice from the ultraviolet to the microwave: A revised compilation. *Journal of Geophysical Research Atmospheres*, 113(14):1–10, 2008.

[4] S. Fukusako. Thermophysical properties of ice, snow, and sea ice. *International Journal of Thermophysics*, 11(2):353–372, 1990.

[5] Aaron Himmler. *Improved EPR Detection at Low- Temperature DNP Conditions*. PhD Thesis, ETH Zürich, Zurich, 2023.
